# Alliances and competitions between nematodes and other micro-organisms occurring in the soil biosphere: a review

**DOI:** 10.3389/fpls.2026.1921015

**Published:** 2026-09-03

**Authors:** Giada d’Errico

**Affiliations:** Research Centre for Plant Protection and Certification, Council for Agricultural Research and Economics, Battipaglia, Italy

**Keywords:** bacteria, fungi, plant-parasitic nematodes, soil biodiversity, viruses, yeasts

## Abstract

The soil biotic community is characterized by many interactions and mechanisms that drive essential soil functions and prevent the outbreak of pests and pathogens. However, the impacts of global environmental changes and intensive agricultural practices on the microorganism diversity remain a critical gap. The decline in microbial diversity could severely affect nematode diversity and their population densities, compromise soil health, and consequently crop productivity. However, an updated, coomprensive and recent review is not available. Therefore, I provide a comprehensive review to highlight the importance of soil biosphere interactions, discuss the main neutral, negative and beneficial interactions between nematodes and other organisms as bacteria, fungi, yeasts and viruses. Also, the review suggests future researches and sustainable strategies in crop protection. Maintaining soil health and safeguard soil biodiversity is fundamental to limit nematode densities and for sustaining agricultural productivity.

## 1 Introduction

The soil ecosystem is complex, heterogeneous, and dynamic. Natural processes are the result of multiplex interactions between biotic and abiotic components of the ecosystem. Biotic component is composed by several living organisms, including a large number of nematodes and microorganisms, such as bacteria (also actinomycetes), fungi, microalgae, and protozoa, as well as earthworms and invertebrates. It is estimated that 1 gram of fertile soil may contain several billions of microbial cells. The composition of microorganisms in the soil biosphere is reported in **Table 1** (Alexander, 1977; Lynch, 1982). These numerous and diverse microorganisms exhibit a different array of interactions (alliances or competitions) among each other and plants, playing important roles (ecological and ecosystem services) in soil life and plant performance. Until now, the fertility of the great majority of cultivated fields on our planet has been increasingly depleted in the pursuit of larger and more profitable yields. This situation has been caused mainly by the excessive and not always justified use of chemicals coupled with monoculture. In turn, this has led to an increasing dependency of agriculture on the constant application of chemicals that in turn has reduced biodiversity and thus resulting in various negative impacts on the environment (Oka, 2020) and crop yield. Yield losses caused by different plant parasites and parasitic weeds (Kranz, 2005; Döring et al., 2011; Savary et al., 2012) are estimated to average 10-40% per year (FAO, 2021). The different interactions among soil organisms may involve more components with direct or indirect beneficial, neutral, or negative effects on nematode and crop yield. Generally, they can be mutualistic, helping each other to survive and reproduce, or antagonistic, competing for food and space (Topalovic and Heuer, 2019). Moreover, soil microorganisms are responsible for basic ecosystem processes, such as the degradation of soil organic matter (SOM). They play a crucial role in maintaining stability of the food network and facilitating nutrient cycles. Differences in the structure of microbial crop plant growth communities are the cause of variation in bio-geochemical processes (Bahram et al., 2018; Crowther et al., 2019). Some microorganisms are able to stimulate plant growth and development, promote nutrient absorption and bioremediation, and increase soil microbiome diversity. Conversely, a relatively small proportion of microorganisms may cause economically significant damage to crop plants. Usually, these beneficial organisms tend to live in proximity to the plant roots (rhizosphere microbiome). The recruitment of beneficial microbes might be crucial under environmental stress conditions to overcome biotic and abiotic stresses (Zamioudis and Pieterse, 2012). During the last decades, one of the most important achievements of scientific research is the increasing documentation of various processes and functions that characterize soil-plant ecosystems, and how microorganisms (including soil meso- and macro-fauna) drive the evolution and sustainability of them to ensure soil and plant health and protection (Zarb et al., 2025).

**Table 1:** Microrganisms living in the soil biosphere.

Organism	Number	
	m^2^	g
bacteria	10^13^ - 10^14^	10^8^ - 10^9^
actinomyceta	10^12^ - 10^13^	10^7^ - 10^3^
fungi	10^10^ - 10^1 1^	10^5^ - 10^6^
microalgae	10^9^ - 10^10^	10^3^ - 10^6^
protozoa	10^9^ - 10^10^	10^3^ - 10^5^
nematodes	10^6^ - 10^7^	10^1^ - 10^2^
earthworms	30 - 300	-
other invertebrates*	10^3^ - 10^5^	-

* Arthropods (shellfish and insects), ascarids and molluscs.

Several researchers have ascertained that soil micro-organisms diversity may greatly reduce the ability of pests or pathogens to colonize the soil (Bender et al., 2016), resulting in the containment of plant-parasitic nematode (PPN) populations (Elhady et al., 2017; Elhady et al., 2018). These soils, known as suppressive soils (Romanenko et al., 2008), affects also nematodes by mechanisms not yet fully understood, involving different biological processes (Devi and Meetei, 2018) in which a given plant pathogen or pest species reduces its ability to reproduce in response to soil microbiome (Bent et al., 2008; Adam et al., 2014; Dutta et al., 2019; Topalović et al., 2020). Plant infestations by PPNs may affect the composition and diversity of the soil microbial community (Hussain et al., 2018; Neilson et al., 2020; Giffard et al., 2022). Some negative impacts on soil ecosystem may also occur because of agricultural practices that can affect the soil’s physical and chemical properties and consequently biological factors (Habteweld et al., 2022). In suppressive soils, plant pathogens or pests do not cause significant damage for several reasons (i.e., difficulties in ecosystem establishment). This suppressivity can be general, if due to the activity of the microorganisms present in the soil community, or specific if due to a single antagonist or a single microbial group (Cook, 2014). Although soil suppressiveness constitutes a biological phenomenon that is difficult to quantify directly, it represents a crucial parameter for both farm economics and environmental quality. Regarding general suppressiveness, a major contribution is provided by the input of organic matter. Beyond its established efficacy in suppressing nematodes through toxic decomposition products (Linford et al., 1938), organic matter serves multiple functions, particularly the preservation of the biodiversity required to sustain a temporally stable food web (Wagg et al., 2014). A frequently cited example of general suppressiveness is the protection of wheat plants against the fungal pathogen *Gaeumannomyces graminis* var. *tritici*. On a global scale, this disease occurs under wheat or barley monoculture regimes. The suppression of this pathogen is mediated by the production of 2,4-diacetylphloroglucinol, a compound exhibiting broad-spectrum antibiotic activity against various phytopathogenic species. This substance is synthesized by beneficial bacteria belonging to the genus *Pseudomonas*, whose abundance increases over successive years of monoculture (Kwak and Weller, 2013). Conversely, specific suppressiveness is highly targeted, with the suppressive properties of the soil being effective exclusively against distinct targets. For instance, non-pathogenic fungi of the genus *Fusarium* residing in the soil can significantly inhibit the fungal pathogen responsible for tomato (*Solanum lycopersicum*) Fusarium wilt, thereby safeguarding the plant from infection. This specific pathogen-inhibiting activity is associated with the production of peptaibols by beneficial *Fusarium* species, which act as membrane-active antibiotic compounds (Larkin and Fravel, 1998). Numerous instances illustrate this phenomenon; notably, the interactions between various strains of certain *Trichoderma* species, particularly *T. harzianum*, *T. virens*, and *T. viride* and nematodes, especially those within the genus *Meloidogyne*. These fungi operate via mechanisms such as mycoparasitism, antibiosis, competition for space in the rhizosphere, production of lytic enzymes, and modulation of plant defense responses. They also synthesize metabolites that impair nematode motility, reproduction, and survival, including gliotoxin, viridin, and cyclosporin A. Furthermore, they secrete enzymes such as chitinases, proteases, lipases, and glucanases, which degrade both the nematode cuticle and its eggs. Additionally, *Trichoderma* spp. induce a systemic response in plants by modulating phytohormones, including jasmonic acid, ethylene, salicylic acid, and auxins (Contreras-Soto et al., 2025). Consequently, suppressive soils possess remarkable to maintain soil functionality, safeguarding biodiversity conservation.

## 2 Soil microrganisms and their interactions

### 2.1 Nematodes

**Figure 1:**
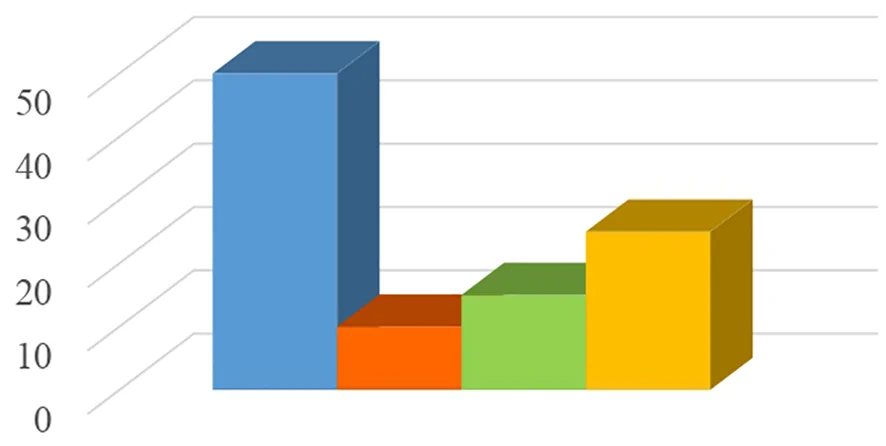
Percentages of nematodes in different habitats (Ravichandra, 2008): marine free-living nematodes (blue), soil and fresh water free-living nematodes (orange), zooparasitic nematode (green) and plant-parasitic nematodes (yellow).

Nematodes live in all kinds of environments wherever life is possible, including salty habitats (Howard and Platt, 1985) and freshwater (Pérez-García et al., 2019). They are also reported from extremely difficult environments, such as polar (Dzido et al., 2009) and desert (Sapir, 2021) regions. They can be parasites of animals, including humans, free-living, and also plant parasites. The composition of diverse nematode groups in different habitats is reported in **Figure 1**.

Of the about 28.000 known species, 16.000 parasitize animals, insects, and plants (Hugot et al., 2001), of which 4.100 species are phytopathogens (Jones et al., 2013) and potentially dangerous to crop plants (Elling, 2013). The ecological success of nematodes is due to the property of the nematocenosis of being composed of large numbers of specimens (several million per m³ of soil), which is much superior to that of other taxa (Gerlach, 1971; Vincx, 1989). They represent the largest group among the metazoa, of which they make up more than 80% (Stöckli, 1946).

Soil nematodes contribute to regulating the biogeochemical cycle and to strengthening vegetation dynamics (van den Hoogen et al., 2019). They also play important roles in the biological chain as energy consumers and suppliers. In fact, several species feed on fungi and bacteria, while others feed on protozoa, tardigrades, oligochaetes, other nematodes, arthropods, or are parasites of insects (entomopathogenic nematodes). Moreover, many species are energy suppliers for viruses, bacteria, protozoa, tardigrades, nematodes, oligochaetes, arthropods, and predatory fungi (Cayrol, 1971). In agricultural systems, nematodes can be subdivided into three groups: free-living nematodes (FLNs) (feeding on bacteria, fungi, other nematodes); entomopathogenic nematodes (EPN), and PPNs (Lambert and Bekal, 2002; Grabau and Dickson, 2018). Based on their feeding habits, nematodes are known as plant feeders, bacterial feeders, fungal feeders, algal feeders, animal predators, and omnivores (Yeates et al., 1993). Besides their feeding diversity, nematodes also vary in their body size and life cycle span.

#### 2.1.1 Nematodes interaction with other nematodes (predator nematodes)

The trophic group of the nematodes involved is that of predators (Khan and Kim, 2007), the most important members of which are species of Mononchina. Most of the information on these nematodes is from experiments on agar, as only limited information has been obtained from field conditions. Nevertheless, a few studies have demonstrated that these nematodes are effective in suppressing soil population densities of several species of PPNs, especially root-knot nematodes (RKN), both in pots and field conditions. The majority of these nematodes belong to the following four orders: Mononchids, Dorylaimids, Diplogasterids, and Aphelenchids, which differ according to their stomatal apparatus, alimentary preferences, and feeding habits (Bilgrami, 1997; Wyss and Grootaert, 1977; Mendoza-de Gives, 2022). These nematodes, besides being useful because of their predation, stimulate plant nutrient cycling and are indirectly a hindrance to the presence of PPNs on the roots (Yeates and Wardle, 1996).

Mononchida have a large and strongly sclerotized buccal cavity provided with several teeth, sometimes also with rows of denticles, used to capture prey. Their feeding apparatus is of the cutting and phagocyting type (such as in *Mononchus, Iotonchus, Mylonchulus*), and if the prey is small, it is ingested as a whole (Banage, 1964; Bilgrami et al., 1986). Besides nematodes, they feed on a variety of microorganisms, including rotifers, tardigrades, small oligochaetes, etc. Unlike other predatory nematodes, they do not immobilize their prey. They are known as cruel killers (Tarjan, 1957) because they are also cannibalistic and are so voracious that a single specimen of *Mononchus* can prey on up to 80 J2 of *Meloidogyne* every day (Steiner and Heinly, 1922). However, they do not tolerate strong environmental changes, have a rather long life cycle, and have low reproduction rates. Their occurrence in intensively cultivated fields is nearly lacking.

Diplogasterids (*Mesodiplogaster*, *Mononchoides*, etc.) have a small buccal cavity but are endowed with a strong dorsal and mobile tooth similar to a claw. Teeth and denticles also help the nematode to tear the prey’s cuticle and grind food particles (Jairajpuri and Bilgrami, 1990). They abound in decaying manure and appear to be more selective than other trophic groups. It has been ascertained that under Petri dish conditions, besides acting as predators, they also behave as bacterivores (Sohlenius, 1973).

Dorylaimids include numerous predatory genera (*Discolaimus, Dorylaimus, Labronema*, etc.). Their stylet is hollow (odontostyle) and serves to pierce the prey and suck its contents. The species of *Sectonema* and *Nygolaimodea* have a large tooth with which they puncture and cut the body of the preyed nematode.

Generally, Aphelenchida are fungivorous, parasites of aerial plant parts, or predators of insects; among them, the genus *Seinura* is the most typical predatory nematode genus. This genus contains small-sized nematodes having a small stylet but able to prey on nematodes larger than themselves (Linford and Oliveira, 1937). The prey, when encountered by chance, is perforated and paralyzed quite immediately by the digesting enzymes that disintegrate the prey’s tissues, thus becoming ready to be sucked; sometimes more predator specimens attack the same specimen of prey (Hechler, 1963).

#### 2.1.2 Nematode-plant interaction

PPNs are the most investigated nematodes because of the severe damage they cause to a wide range of crop plants that include vegetables, flowers, fruit trees, forest plants, and lawns, and they are very important for global food security (Eisenback, 2020). Ten species or groups of PPNs are the most economically important worldwide (Jones et al., 2013). Among them, there are: RKN of the genus *Meloidogyne*; the cyst-forming nematodes of the genera *Globodera* and *Heterodera*; the root lesion nematode (*Pratylenchus* spp.); the pine wood nematode (*Bursaphelenchus xylophilus*); the burrowing nematode (*Radopholus similis*); the rice white-tip nematode (*Aphelenchoides besseyi*); the reniform nematode (*Rotylenchulus reniformis*); the stem and bulb nematode (*Ditylenchus dipsaci*); the dagger nematode (*Xiphinema index*), a virus vector especially of the grape fan leaf virus (GFLV), which is the most economically important virus of grapes; and the false root-knot nematode (*Nacobbus aberrans*). However, the most damaging species are the RKNs, cyst nematodes, and root lesion nematodes (Sikora et al., 2018). The severity of damages caused by PPNs varies according to crop and world geographical area (Oerke et al., 1994). In developing countries, agricultural technologies are scarce or lacking, and food availability is insufficient; thus, the impact of PPNs can be more severe. Worldwide, it is estimated that approximately 12.3% of the crop yield is annually lost (157 billion dollars) (Singh et al., 2015; Mendoza-de Gives, 2022). Estimates of lost production are potentially more critical under protected environments, such as in greenhouses, in which crop plants can be cultivated year-round, and nematodes may co-exist with fungal and bacterial phytopathogens causing negative or synergistic damages (Ragozzino and d’Errico, 2011; Zhang et al., 2020). In such associations, PPNs are considered primary pests favoring the infection and establishment of secondary pathogens (fungi and bacteria) that can penetrate cells through wounds caused by primary pathogens (Mayol and Bergeson, 1970; Powell, 1979). Moreover, symptoms caused by PPNs may reduce yield quality (Palomares-Rius et al., 2017), as well as quantity, when they infest edible plant parts or by viruses transmitted to plants by nematodes belonging to the families Longidoridae (genera *Longidorus, Paralongidorus*, and *Xiphinema*) and Triplonchidae (genera *Trichodorus* and *Paratrichodorus*) (Ciancio, 2014).

Damages caused by PPNs depend on the nematode species, the initial soil population density, its reproduction potential, soil type, the period of the year of the crop cycle, the crop plant, and its susceptibility, tolerance, and resistance in the given agricultural system (Nicol et al., 2011). The challenge of this century consists of fulfilling the food needs of the world’s human population while simultaneously obtaining good quality yield and preservation of the biodiversity of the agricultural agro-ecosystems.

PPNs and FLNs may carry plant pathogens, attached to their cuticle or endogenously, that they transport into the soil rhizosphere or into the roots and stimulate the formation of the pathobiome. On the other hand, it has been suggested that specific rhizobiomes transmitted via the cuticle of endoparasitic PPNs into the roots can protect PPNs against the PPN-antagonistic root endophytes (Brown, 2018; Topalović and Vestergård, 2021). PPNs also make crop plants more susceptible to other plant pathogens (Hunt and Handoo, 2009; Lunt et al., 2014).

#### 2.1.3 Nematode-bacteria interaction

The group of nematodes involved is that of bacterivorous. This is one of the most important and largest in terms of the number of species, including nearly all FLNs in the soil; they are devoid of stylet. These nematodes carry in their alimentary tract a mixture of bacteria they use as nourishment, which are disseminated in substrates where they proliferate, while simultaneously secreting substances that stimulate nematode development (van Haut, 1956; Cayrol and B’Chir, 1973). Such associations allow these nematodes to play an important role in soil life as they spread and keep under control primary decomposers. By spreading in substrates, bacteria can be noxious or useful to plants indirectly affecting plant development. An example is given by the nematode *Rhabditis lambdiensis*, which stimulates the multiplication of the bacterium *Pseudomonas tolaasii*, the causal agent of the bacterial spot in the cultivated edible mushroom *Agaricus bisporus* (Steiner, 1933; Brodey et al., 1991). These nematodes may become directly damaging to crops, accelerating plant senescence and death by speeding the decay of roots severely infected by PPNs, especially RKNs.

This group also includes entomopathogenic nematodes (EPNs) of the families Steinernematidae and Heterorhabditidae, which live in symbiosis with bacteria of the genera *Xenorhabdus* and *Photorhabdus*. For instance, *Steinernema feltiae* releases *Xenorhabdus* into the hemolymph of the insect where they multiply rapidly, secreting large numbers of toxins and exoenzymes, and kill the insect by septicemia (Clausi et al., 2014).

Bacteria are unicellular microorganisms that occur in all soil types, in radioactive residues, hot water springs, etc. (Fredrickson et al., 2004; Hussain and Khan, 2020; Hussain et al., 2020) as they are adapted to an array of environmental conditions. They constitute the largest proportion of the microfauna; soil may contain billions of bacteria, and they are more abundant in the soil rhizosphere (rhizosphere effect) than in soil uncovered by vegetation (Lynch, 1982). Bacteria are instrumental for the equilibrium of ecosystems. A number of bacteria of the genera *Bacillus*, *Pseudomonas*, *etc*., known as plant growth-promoting bacteria (PGPB), live in the soil rhizosphere (rhizospheric bacteria) or inside plant roots (endophytic bacteria), greatly stimulate plant growth and development (Borriss, 2011; Shivlata and Satyanarayana, 2017; Khan et al., 2022). This promotion follows different beneficial processes, such as biofertilization, release of phytormones, control of plant pathogens, and induced plant resistance (ISR) to biotic stresses (Lugtenburg and Kamilova, 2009; Aioub et al., 2022).

Bacteria enrich the beneficial microbiota of the soil with a disadvantage for harmful microorganisms and closely interact with the soil biota, especially nematodes; they share the same microhabitat and cooperate or compete in the same ecological roles: some nematodes feed mainly on bacteria. Their populations vary according to soil characteristics, especially soil moisture content and soil temperature, and may increase or decline rapidly. Normally, due to their oxygen needs, aerobic bacteria are abundant in sandy soil and well-drained soil; on the contrary, anaerobic bacteria prevail in clay and wet soils, where they may release substances toxic to plant roots, predisposing them to several diseases. In soil, the presence of many species of bacteria from different genera is very useful, even because they are effective in killing nematodes by implementing sophisticated strategies, eventually used for feeding, thus acting as natural regulators of the soil nematode populations. The most important PGPB are nitrogen fixing bacteria, phitormone-producing bacteria, phosphorous solubilizing bacteria, siderophore-producing bacteria, plant growth promoting rhizobacteria (PGPR). All these bacteria group can be used by bacterivorous nematodes for their feeding which, therefore, reduce their beneficial effect on plant growth. Other groups of bacteria are plant-damaging bacteria, nematophagous bacteria, parasporal bacteria forming crystal proteins and endophytic bacteria. Bacterivorous nematodes may also feed on plant parasitic bacteria thus reducing their negative effects on plants while nemathophagous bacteria may greatly suppress nematodes, and as so are being exploited for their use as biological agent for the control of PPNs. Hereafter we discuss on these three last groups of bacteria.

Nematodes are primary pathogenic agents and therefore favor the aggressiveness of the bacteria that otherwise could not infect plants (Mayol and Bergeson, 1970; Powell, 1979). Such a phenomenon was observed in a split-root experiment with tomato cv. Tropic in the presence of *M. incognita* and the bacterium *Rhizobium radiobacter* (=*A. tumefaciens*) (El-Sherif and Elwakil, 1991). Besides, RKNs play several other useful roles, such as suppressing or modifying host plant defense mechanisms (Smant and Jones, 2011) and the plant immune system (Abramovitch et al., 2006). However, interactions between nematodes and bacteria are rather limited when compared to those between nematodes and fungi. Although with contradictory results, synergistic interactions on several crops, particularly kiwi fruit, cotton, peach, rose, grapes, etc., occur frequently between PPNs and phytopathogenic bacteria, resulting in increased severity of the disease (Pitcher and Crosse, 1958; Pitcher, 1963; Pitcher, 1965). This occurs because PPNs are also primary agents for bacteria, although they colonize only cells killed by primary agents (Mayol and Bergeson, 1970; Powell, 1979). The most frequent interactions are between RKNs and *R. radiobacter* (Rubio-Cabetas et al., 2001; Lamelas et al., 2020). These bacteria were found in the soil for up to 40 years in the absence of a susceptible host (Crosse, 1968). Species belonging to this genus, such as *R. rhizogenes* (=*A. rhizogenes*), which causes only mild disease in roses under field conditions in California, if present concomitantly with the root lesion nematode *P. vulnus*, may cause severe damage. Less common and occurring only in limited areas are the associations between bacteria and other PPNs, especially those infecting the aerial plant parts. In this review, there are mentioned some of the most interesting for their symptoms and damage severity. In India, *Rathayibacter* (=*Clavibacter*) *tritici* is associated with *Anguina tritici*, causing a disease known as “Tundu” of wheat, consisting of the rot of kernels that are very toxic to animals feeding upon them. The nematode J2s are necessary for the development of the disease as they transport bacterial cells into the kernel (Nandal et al., 2010). Of interest is the association between *A. agrotis* and *R. toxicus* on annual ryegrass in south and west Australia, which causes the death of cattle and sheep feeding on this infected graminaceous plant (Bird and Riddle, 1984). *A. fragariae* and *A. ritzemabosi* alone do not cause diseases, but they may interact with *Rhodococcus fascians* (=*Corynebacterium*), causing the cauliflower disease of strawberry that shows short and enlarged floral and leaf stems, giving the inflorescence the appearance of a small cauliflower (Crosse and Pitcher, 1952). The bacteria alone may only cause crowns and secondary galls. The stem and bulb nematode *Ditylenchus dipsaci* can also be associated with the bacterium *Corynebacterium* insidiosum to cause bacterial wilting of alfalfa (Hawn, 1963); in this case, the bacterium is transmitted by the nematode from infected to non-infected alfalfa plants (Hawn, 1971). Damage is caused by a blue compound not soluble in water that accumulates at the extracellular level and is characterized first by yellowing and white leaves and then by wilting and death of the plants. Another association was observed on tomato between *C. michiganense* and *M. incognita*: the RKN increases the severity of the bacterial cancer both on cancer-susceptible and resistant tomato (Moura et al., 1975). Very interesting, for the biological control of insects, are the associations between bacteria and bacterivorous nematodes mentioned earlier.

##### 2.1.3.1 Nematophagous bacteria

They are a large group of deeply investigated bacteria, overall because many of them are natural antagonists of PPNs. They act as parasites by releasing antibiotics and enzymes, inducing genetic resistance, competing for nutrients, and thus improving plant health. Their beneficial effects may occur by acting directly on the nematodes or indirectly through the improved plant health, colonization of the plant rhizosphere, or through microbial activity. Based on the mode of action, they can be obligate parasites, opportunistic parasites, rhizobacteria, parasporal producing crystal proteins, endophytes, and symbionts (Tian et al., 2007). Except for the obligate parasites, nematophagous bacteria usually live as saprophytes, and nematodes may constitute just a possible food source. This vast group of bacteria is widely distributed and has a large number of hosts; they have been isolated from the soil, plant tissues, and from nematodes, including eggs and cysts. The obligate parasites are well represented by a large number of Gram- positive bacteria of the genus *Pasteuria* from the family Bacillaceae. They are specific antagonists, produce infective and long-lasting endospores provided with parasporal fibers similar to cups, capable of sporulating within adult (Sayre et al., 1991), juvenile (Sturhan et al., 1994), or both nematode stages (Ciancio, 1995; Galeano et al., 2003). The typical representative of this genus is *P. penetrans*, a bacterium well-known for having a large variety of forms and nematode hosts, especially RKN (Stirling, 1991). Thereafter, several other species have been found (*P. thornei, P. nishizawae, P. usgae, P. hartismeri*) on nematodes from different trophic groups. Endospores produced by *P. penetrans* are very resistant to high temperatures, drying, and to chemically active ingredients. Infection occurs passively by adhesion of the endospores to the nematode cuticle while moving in the soil, followed by penetration into the nematode, development within it (parasitic phase), and decay of the nematode content due to bacterial multiplication and fragmentation of its dichotomous thallus that invades the nematode. The parasitic cycle of the bacterium ends with the formation of new endospores that fill the nematode body and diffuse into the soil after nematode death. Opportunistic bacteria may shift from saprophytic to parasitic habits, and nematodes are just a possible food source. This bacterial group penetrates the nematodes through the cuticle and then kills the host. The mechanism of predation, deeply investigated in *Brevibacillus laterosporus* strain G4, has shown that these bacteria, after attaching to the nematode cuticle, can multiply to form clones that, following the cuticle degradation and ingestion, form a hole that allows bacteria to enter the host body and digest internal tissues (Huang et al., 2005). The most representative of this group, known for being useful biocontrol agents, are two species of the genus *Bacillus*: *B. subtilis* and *B. firmus. B. subtilis* is a Gram-positive aerobic and opportunistic pathogen forming endospores (Boer and Diderichsen, 1991). Differently from other species, *B. subtilis* is classified as an obligate aerobe that can survive as quiescent endospores even for many years. Endospores of other nematophagous species under environmental and nutritional stress conditions increase their survival in the rhizosphere (Rao et al., 2017). Surprisingly, endospores were found to survive ingestion by bacterivorous microorganisms, such as the protozoan Tetrahymena thermophila and the nematode *Caenorhabditis elegans* (Klobutcher et al., 2006; Carroll et al., 2008), and sporulation begins when nutrients are exhausted. *B. firmus* is an aerobic Gram-positive bacterium naturally occurring in the rhizosphere and is considered one of the most promising biocontrol agents for RKNs (Stirling, 1991). It has a double mechanism of action: direct on nematode eggs, which are killed by the toxic metabolites, and inhibited in their hatching even if protected inside the gelatinous matrix of the egg masses (Keren-Zurr et al., 2000; Mendoza et al., 2008), and indirect through the surface colonization of the roots, creating a physical barrier that impedes the establishment of nematodes and degrading root exudates that lose their attractiveness to phytopathogens. The most studied strain is *B. firmus* I-1582; it was isolated from soil in Israel and is known to be reproduced in large scale and formulated for application under field and protected conditions. Rhizobacteria comprise numerous genera occurring in the soil (*Agrobacterium, Arthrobacter, Azotobacter, Beijerinckia, Clavibacter, Rhizobium*, etc.), of which some strains of *Bacillus* and *Pseudomonas* are known for their nematophagous activity (Marin-Bruzos and Grayston, 2019; Aioub et al., 2022). Their mode of action can be direct, through the production of toxins, enzymes, and other metabolic products (Siddiqui and Mahmood, 1999), or indirect by regulating nematode behavior (Sikora and Hoffmann-Hergarten, 1993) through plant-nematode recognition (Oostendorp and Sikora, 1990), the production of chemicals, substances useful to plants (Backer et al., 2018), and induction of systemic resistance (Hasky-Günther et al., 1998). The activity of the microbial community, as for rhizobacteria, is stimulated following soil application of manure or other organic soil amendments (Giannakou and Prophetou-Athanasiadou, 2004).

##### 2.1.3.2 Parasporal bacteria forming crystal proteins

Parasporal bacteria forming crystal proteins (Cry) include the species *B. thuringiensis* (Bt), which was identified more than 1 century ago in Japan from larvae of the silkworm (*Bombyx mori*) causing sudden death (Ishiwata, 1901). In some countries, it was used soon as a biocontrol agent due to the lack of synthetic nematicides; however, the commercial product was released in France only in 1938 (Aronson et al., 1986). Bt is a Gram-positive spore-producing bacterium that lives in agricultural soils, on leaf surfaces, in water environments, in animal feces, in milling establishments, and in wheat stores (Madigan and Martinko, 2005). Of the dozen subspecies described, *B. thuringiensis kurstaki* (Btk) is the most known. The activity toward insects of this bacterium occurs because of the crystal proteins (Cry or δ-endotoxins) (prototoxins) contained in the microbial spores. These toxins cause deep alterations in the host that lead to paralysis of the intestine and of the muscles of the digestive apparatus, but not of the nervous system, as occurs instead with the most common synthetic insecticides. The death of the prey occurs in a rather short time, varying from 3 to 5 days. Besides lepidopterans, Bt attacks also coleoptera, diptera, hymenoptera (Maagd et al., 2001) and some invertebrates such as mites, protozoa, and especially nematodes (Feitelson et al., 1992; Yu et al., 2015; Wan et al., 2019). On nematodes, the Cry proteins, particularly Cry5, Cry6, Cry14, Cry21, and Cry55 (Wei et al., 2003; Ali et al., 2021), act by forming lytic pores in the cellular membrane of the intestinal epithelial cells (Crickmore, 2005; Shi et al., 2020). The toxicity of Cry is toward epithelial cells of the midgut and leads to the formation of pores, vacuoles, depressions, and complete intestinal degradation Marroquin et al., 2000). Bt plays a number of roles similar to those of other bacteria, but overall it favors systemic immunity, thus keeping parasites and pathogens under control and therefore increasing crop yield (Gupta et al., 2024).

##### 2.1.3.3 Endophytic bacteria

Endophytic bacteria occur in all plant parts (Surette et al., 2003; Qin et al., 2009; Compant et al., 2011; Elmagzob et al., 2019; Harrison and Griffin, 2020), with which they establish different relationships such as symbiotic, mutualistic, commensalistic, and trophobiotic. The majority of the endophyte bacteria appear to derive from the rhizosphere or phyllosphere and a certain amount from seeds. Phytophagous insects and pathogens of crops are responsible for severe economic losses in agriculture; it has been demonstrated that a correlation exists between bioactive compounds produced by endophyte bacteria and the tolerance to phytophagous insects and pathogens of the host plant (Bibi et al., 2012). Bacteria of the genera *Arthrobacter, Pseudomonas, Serratia, Bacillus*, and *Curtobacterium* (Aravind et al., 2009; Aravind et al., 2010) are among the best representatives of bacteria used for the biocontrol of plant pathogens. Besides, these bacteria are very important in improving plant growth and increasing yield, inducing systemic resistance, in soil bioremediation, and increasing soil fertility through solubilizing phosphates and fixing atmospheric nitrogen (Compant et al., 2005; Ryan et al., 2008). Some rhizobacteria may also increase the resistance of the host plants to abiotic stresses, such as heavy metals and salinity (Sheng et al., 2011), and increase the biomass of roots and shoots while stimulating seed germination (Vendan et al., 2010). Endophyte bacteria and pathogen microorganisms occupy the same niche within the plant, and therefore, inoculation of these bacteria into plants is a sound method for the biological control of pathogens (Senthilkumar et al., 2011). However, biotic and abiotic factors do affect the community of these bacteria, such as species composition, structure of the communities, diversity, and functions (Walitang et al., 2018).

The symbiotic bacteria represented by *Xenorhabdus* and *Photorhabdus* (Enterobacterales: Morganellaceae) are so closely related phylogenetically that, in the past, they were considered to belong to the same genus (Thomas and Poinar, 1979). They are symbionts of the entomopathogenic nematodes (EPN) *Steinernema* and *Heterorhabditis*, in particular *S. feltiae, S. carpocapsae, S. kraussei, H. bacteriophora* and *H. megidis*. The symbiotic relationship between nematodes and bacteria allows the nematodes to protect the bacteria from the external environment and to be introduced into the host, while the bacteria provide the nematodes with the food necessary for their development. The insects are infected by the third-stage juveniles (IJ) of the nematode, the only nematode stage occurring in the soil, which enter the insect through natural openings (stigmas, anus, buccal cavity) (Triggiani and Poinar, 1976) or through intersegmental openings (Bedding and Molineux, 1982; Peters and Ehlers, 1994) in the larvae of the host insect, releasing the symbiotic bacteria (*Xenorhabdus* spp. for the Steinernematidae EPNs and *Photorhabdus* spp. for the Heterorhabditidae EPNs) into the hemocoel of the victim, which is killed within 24–48 hours (Akhurst and Boemare, 1990; Dillman et al., 2012). In the hemocoel, bacteria produce cytolysins, hemolysins, and toxins, some of which induce apoptosis or necrosis in the host cells (Nielsen-LeRoux et al., 2012), leading the host tissues to form a kind of culture broth (Simoes et al., 1992) and release antibiotic substances to avoid putrefaction, thus favoring the development of 2–3 generations of the nematodes (Akhurst, 1990). When all food is exhausted, the IJ exits from the prey in search of a new host insect. Larvae of scarabaeids and tenebrionids escape anal penetration by the nematode by doing frequent defecations (Forschler and Gardner, 1991). This symbiotic complex has also shown an antagonistic effect toward several species of PPNs, particularly RKNs, and the pinewood nematode *B. xylophilus* (Hu et al., 1999; Fallon et al., 2004; Lewis and Grewal, 2005), which is vectored exclusively by cerambycid coleoptera, mainly some species of the genus *Monochamus*. Recent studies suggest that the bacterial symbionts of the EPNs, especially *Xenorhabdus*, are very interesting agents to be used as biological nematicides for the control of PPNs (Tomar et al., 2022). *Xenorhabdus* bacterial species, after entering the host, release secondary nematicidal metabolites that kill it, suggesting that these bacteria may represent a significant biological control mechanism against RKNs (Caccia et al., 2018; Abebew et al., 2022).

#### 2.1.4 Nematodes-fungi interaction

Fungivorous nematodes include several genera, especially *Aphelenchus*, *Aphelenchoides*, *Ditylenchus*, *Bursaphelenchus*, etc., belonging to the order Tylenchida. A few species, such as *D. myceliophagus*, known as the “nematode attacking the mycelia of cultivated mushrooms,” may play the dual role of being useful or deleterious to crop plants: while being one of the most damaging nematodes of cultivated mushrooms, particularly *A. bisporus*, it also feeds and reproduces on a number of soil fungi, including phytopathogenic fungi, acting as a biocontrol agent. Conversely, *A. avenae* may impede the establishment of the symbiosis between endomycorrhizal fungi of the genus *Glomus* and plant roots. Because of this, it is suggested that the soil be disinfected with nematicides, preferably fumigants, before transplanting small pine trees. Soil fumigation, besides improving the establishment of useful microorganisms applied to the soil, creates a biological vacuum (d’Errico et al., 2022), favoring the mycorrhizal infection of the pine roots and allowing this conifer to use dead nematodes as an excellent organic substrate (Sutherland and Fortin, 1968; Bakhtiar et al., 2001; Ragozzino and d’Errico, 2011). A few species of fungivorous nematodes in the genus *Labronema* also occur in members of the Dorylaimida nematodes (Ferris, 1968). Fungivorous nematodes include species having a thin stylet in the stoma, sometimes without basal knobs, and feed exclusively on fungal mycelium and, exceptionally, as observed in *Eudorylaimus ettersbergensis*, on conidia (Hollis, 1957). In the soil, contact between nematodes and fungi occurs by chance, as, unlike plant roots, fungi do not secrete root exudates attractive to nematodes (Anderson, 1964: Cayrol and Ritter, 1967). Penetration of the nematode stylet into the fungal hypha occurs perpendicularly, and the ingestion of its contents is prompt and rapid, as it occurs with *D. myceliophagus*, which sucks in the amount of cellular protoplasm required to fill its pharynx in about 30 seconds, or takes longer (up to 3 hours) in species whose ingestion of cellular protoplasm is preceded by the injection into the fungal cell of secretions from the dorsal esophageal gland of the nematode, allowing for pre-digestion before ingesting cellular content. It should be noted that a given fungivorous nematode species feeds on only a specific fungal species (Mankau and Mankau, 1963; Cayrol, 1967). Additionally, the selection of the fungus to attack depends on the size of the nematode and the fungal hypha (Cayrol, 1970); nematodes only attack hyphae whose diameter is within 1/3 to 1/4 of the nematode diameter, and this feature must be considered in biological control to select the target.

Fungi represent one of the most important components of the microbiota in many ecosystems (Bahram et al., 2021), sharing the micro-environment, degrading secondary metabolites, and establishing relationships with other groups, such as mutualism, competition, and predation. Such interactions are fundamental for maintaining ecosystem equilibrium (Topalovic and Heuer, 2019). The interaction of fungi with other organisms in the soil biosphere plays a pivotal role because to infect their hosts and access nutrients, fungi employ a variety of biochemical and mechanical strategies, and for their virulence, they produce enzymes that degrade secondary metabolites (Doehlemann et al., 2017). Moreover, some phytopathogenic fungi produce mycotoxins that, remaining in the edible plant parts, can be poisonous to both humans and animals. The composition and activities of fungi are regulated by soil biotic (plants and other microorganisms) and abiotic (pH, soil moisture content, salinity, soil texture, temperature) factors (Lopez-Bucio et al., 2015; Rouphael et al., 2015). Most fungal species interact closely with both living and dead organisms, particularly plants. As they can produce a large number of different extracellular enzymes, they degrade organic matter and regulate carbon and nutrient equilibrium (Žifčáková et al., 2016). Many species adsorb and accumulate toxic heavy metals (lead, copper, zinc, etc.) in their organs, which, however, can be deleterious to them (Baldrian, 2003). Other species play important roles in the soil, such as parasitism, thus becoming the causal agents of severe plant diseases (Li et al., 2017) or can be beneficial as antagonists of nematodes (Mendoza-de Gives, 2022), acting as bio-agents for the biological control (BCA) of nematodes and thus being effective in reducing damage caused by these parasites (Sikora et al., 2008; Van Ooij, 2011). During the last decade, due to the reduction of the availability of synthetic fungicides in the market, greater aggressiveness of pathogenic populations, and global warming, different pathogens have become increasingly damaging, thus lowering the growth of sustainable agriculture. Numerous trophic groups of fungi play important roles in the soil, such as organic matter recycling, production and depletion of energy, food source, parasitism of plants, BCA, and producers of mycotoxins. Hereafter is a presentation of the trophic groups: saprotrophy, plant parasites, nematophagous fungi, and mycotoxin producers and their interaction with nematodes highlighted.

##### 2.1.4.1 Saprotrophic fungi

Saprotrophic fungi (SF) play crucial roles in the functioning of the ecosystem. They are the main group of soil microorganisms degrading soil organic matter, including lignocellulose derived from the cell walls of plants (the main components being cellulose, hemicellulose, and lignin), which is the most resistant to degradation and the greatest renewable organic resource on Earth. They also promote the recycling of nutrients useful for their growth and the development of plants and other organisms (A’Bear et al., 2014a; A’Bear et al., 2014b). A number of scientific articles report that their role in the carbon and nutrient cycle is instrumental (Averill et al., 2014; Fernandez and Kennedy, 2016; Žifčáková et al., 2016; Netherway et al., 2021); at the same time, they are a good source of energy for suppliers (for invertebrates, collembola, and nematodes) and also consumers of energy derived from insects, mites, nematodes, rotifers, etc (Cannon and Kinsey, 1996). Several saprotrophic fungi, particularly some species of the genus *Pleurotus*, are beneficial in adsorbing heavy metals (Mohamadhasani and Rahimi, 2022). Moreover, several species of these fungi, in the presence of prey, may change their lifestyle, shifting from saprophyte to parasite. However, their interaction with nematodes is of limited importance.

##### 2.1.4.2 Phytopathogenic fungi

Phytopathogenic fungi (PF), in particular species of the genera *Fusarium, Rhizococtonia, Botrytis* and *Pythium*, represent a severe menace for agriculture productions at global level (Li et al., 2017) even because the extent of the damage they cause is continuously increasing since the mid of the past century (Savary et al., 2019). Yield losses, despite the numerous fungicides used for control during the crop cycles, have been estimated to vary from 10 to 23% and an additional 10-20% loss occur during the post-harvest period (Fisher et al., 2012). Generally, scientists forecast that damages are increasing because, following the global climate changes, PF infections are moving towards the poles, enlarging their area of diffusion. This means that many more Countries will experience more presence or more intensive presence of PF damaging their crops. Often the damages to crops are not caused by a single pathogen, but they are the result of concomitant infection of two or more of them (Chatterjee et al., 2016). In the evolution history of plants and their PF have emerged very specialized and complex relationships thus creating a model of mutual selection and co-evolution with plants (Quintanilha-Peixoto et al., 2022). Despite some nematodes may feed on PF.

##### 2.1.4.3 Nematophagous fungi

Nematophagous fungi (NF), are the main natural antagonists of PPNs and comprise more than 700 species (Van Ooij, 2011). Their interaction with nematodes is of paramount importance as several species of them can be used as BCA for the management of nematodes. These NF are more abundant in undisturbed soils and have evolved utilizing nematode as a primary food source. They represent an ancient a diversified group that, under unfavourable conditions, use their conidia or mycelial structures to entrap living preys that are used as a food (Nordbring-Hertz et al., 2011), shifting from saprophitic to carnivorous behaviour (Herrera-Estrella et al., 2016). NF have very complex infection habits and are natural predators of nematodes (Braga and Araújo, 2014; Degenkolb and Vilcinskas, 2016). The first fungus described as having nematophagous activity and deeply investigated worldwide as a promising bioagent for the biological control of nematodes is *Arthrobotrys oligospora*. At preying, the prey may react by reducing its vital activity (feeding, reproduction and motility). A recent study, using randomly collected samples, has shown that nematodes and NF occur everywhere and live in the same area in more than 60% of the samples (Yang et al., 2020). In this respect, the nematode *C. elegans* and the fungus *A. oligospora* often coexist, utilizing different mechanisms to trap the nematode (Ulzuurrun and Hsueh, 2018), such as the production of VOCs, which interfere with sex differentiation and alimentary signals (Hsueh et al., 2017). At preying, the fungus causes a mechanic stress inducing quiescence (Lin et al., 2024). To prey, *A. oligospora* forms three-dimensional adhesive nets (Ulzuurrun and Hsueh, 2018). NF because of their high ability to capture and kill nematodes, are investigated not only for their effective nematode biocontrol but also as plant growth promoters because of the increased nutrient availability (Monteiro et al., 2018). The way used by NF to prey nematodes is reported in many reviews (Soares et al., 2018; Gajendiran and Vijayarangan, 2020; Elkhateeb et al., 2023; Vera-Morales et al., 2023; Berhanu et al., 2024). In general, these fungi can be grouped as: predators using specialized hyphal devises to capture and trap nematodes and endoparasites that infect nematodes by their endospores. In such associations, the relationships established between them do not differ from all associations between predator fungi and preys. As a defence mechanism, fungi may produce toxic metabolites (Tayyrov et al., 2018). An example is given by the fungus *Coprinopsis cinerea*, which produces on the surface of its mycelium a bacterial toxin, similar to cytolisine (Tayyrov et al., 2019), that by contact may kill nematodes (Schmieder et al., 2019). Recently, these fungi have been classified in four categories (Berhanu et al., 2024) as follow: nematode trapping fungi (predacious/predatory fungi), endoparasite fungi, parasite fungi that feed on eggs and vermiform females, and toxin producing fungi.

##### 2.1.4.4 Predators or nematode-trapping fungi

Predators, or nematode-trapping fungi (NTF), are an interesting category of carnivorous microorganisms able to produce in the medium nematode-trapping predatory devices such as adhesive nets and adhesive nematode constricting rings specialized to attract, trap, and digest nematodes (Barron, 1979; Moosavi and Zare, 2012). In particular, the species of the genus *Nematoctonus* are not host-specific; therefore, they capture all species of nematodes living in the soil. Up to 200 species described are common in both terrestrial and aquatic ecosystems (Nordbring-Hertz et al., 2011). In general, these fungi can be found in fresh and wet habitats and at rather high elevations (Koziak et al., 2007). For instance, the genus *Pleurotus*, thanks to adhesive substances secreted on the hyphae, captures nematodes passing by and then produces a tube entering the host, and then secretes enzymes that kill the nematode, which in turn is used by the fungus as nourishment. *Nematoctonus* fungi capture nematodes by means of adhesive mycelial traps or adhesive spores shaped like clepsydras surrounded by a thick mucous sheath and not by hyphae (Braga and Araújo, 2014). These traps capture nematodes by sticking during contact (Koziak et al., 2007; Barron, 2004; Luo et al., 2006; Li et al., 2017). The most effective traps are the constricting rings composed of three cells that, when the prey enters, swell, thus quickly immobilizing the prey (Stirling, 1991; Viaene et al., 2006; Lopez-Llorca and Jansson, 2008). Nematode-capturing structures may vary even within the same fungal genus, as observed in *N. robustus* that produces sticking apophyses on the hyphae, *N. leptosporus* on germinating conidia, and *N. angustatus* on both hyphae and conidia (Koziak et al., 2007). A few species produce toxins to kill nematodes (Li et al., 2000).

##### 2.1.4.5 Endoparasitic fungi

*Endoparasitic fungi* (ENF) are obligate parasites of a large array of nematodes that complete their entire life cycle inside the host (Morton et al., 2004; Lòpez-Llorca et al., 2008). These fungi infect soil PPNs using a variety of spores (conidia and zoospores), which, when ingested by the nematodes, germinate in the gut (usually the esophagus or intestine) to complete their life cycle, or their zoospores adhere to the nematode cuticle (Dijksterhuis et al., 1991; Tunlid et al., 1992; Liu et al., 2009), usually around natural openings, to become cysts. Once in the nematode body, these zoospores produce hyphae giving rise to sporangia containing zoospores (Viaene et al., 2006). In *Drechmeria coniospora*, the spores adhere to the cuticle of several species of PPNs (Jansson et al., 1985). Besides, several metabolites have been isolated from ENF. In particular, from the endoparasite *Verticillium chlamydosporium*, a polyketide (fomalactone) has been isolated, which has shown nematicidal activity toward the root-knot nematode *M. incognita* (Khambay et al., 2000). In addition, several aurovertines, aurovertines F and D, isolated from *Pochonia chlamydosporia*, have shown toxicity toward the free-living nematode *Panagrellus redivivus* (Niu et al., 2010).

Parasitic fungi feeding on nematode eggs and vermiform females produce modified hyphae called traps (appressoria or zoospores) with which they stick to and digest nematode eggs, cysts, and females by a mechanical/enzymatic process (Lòpez-Llorca et al., 2008). In the absence of the host nematode, these fungi can survive in the rhizosphere as saprophytes; they are effective in infecting the host nematode stages that are in the substrate (eggs, young developing stages, females) or that cannot move, such as those inside the gelatinous matrix of RKNs and *Tylenchulus semipenetrans*, from which several opportunistic fungi have been isolated (Kerry, 2000). Generally, fungi parasitizing cysts and eggs of nematodes are more numerous than those infecting nematode females (Gintis et al., 1983). This trophic group of fungi is the most investigated for use as BCA. *P. chlamydosporia* and *Paecilomyces lilacinus* are the species most frequently isolated (Siddiqui and Mahmood, 1996). Also the genera *Haptocillium* and *Hirsutella* are considered effective BCA of PPNs (Tribe, 1980; Kerry, 1989).

Fungi producing toxins immobilize nematodes, penetreting the nematode cuticle, and colonizing the nematode body. Before the hypha crosses the nematode cuticle, toxins secreted by the fungi paralyze the nematode. Among these fungi, there are species of the genus *Pleurotus*, mainly *P. ostreatus* (oyster fungus), which are edible mushrooms producing letal toxins as defense mechanism against nematodes (Thorn and Barron, 1984; Genier et al., 2015). The synthesized toxin, trans-2-decenoic acid, called “ostreatin” (Kwok et al., 1992), is secreted at the extremity of swellings on the hyphae (Siddique and Grundler, 2018; Satou et al., 2008) as small drops. The contact with the toxin drop cause the paralyze of the nematode, and once dead, it represents an ideal food source for the fungus (Barron and Thorn, 1987; Pineda-Alegría et al., 2017; Singh et al., 2019). Recently, other compounds have been described as toxins produced by *P. ostreatus*, identified as a protein similar to ribotoxin (Landi et al., 2020) or the protein “ostreolisine” (Žu žek et al., 2006). *P. ostreatus* is very effective against an array of nematodes (Lee et al., 2020). Nematicidal activities have been described for more than 200 species of fungi belonging to the genera *Arthrobotrys, Coprinus, Harposporium, Climacodon, Lentinus, Pleurotus*, and others (Moosavi and Zare, 2012; Jaffee, 2004; Truong et al., 2007; Bellettini et al., 2019). It has also been reported that the nematicidal properties of the mycelium of *P. ostreatus* should not be tested solely for the nematicidal potential of *P. ostreatus*. An investigation on the nematicidal potential of *P. ostreatus* on the nematode *C. elegans* has shown great diversity in the activity of the progeny, in which five eukaryotic mycelia were identified as effective in suppressing populations of PPNs (Kudrys et al., 2022). An additional positive aspect of the potential use as bioagent is the bio-degradability of fungal toxins (Kwok et al., 1992; Satou et al., 2008). It is also important to mention the fungus *Climacodon septentrionalis* causing the white rot in living hardwoods for the production of toxins that paralize *Aphelenchoides* spp. which are destroyed thereafter.

It must be underlined that there are many other species of fungi producing toxins that act on nematodes. Li et al. (2007) analysed 179 different compounds of various chemical groups having nematicidal activity, mainly isolated from deuteromycetes, ascomycetes and basidiomycetes. However, only a small number of nematophagous fungi produce toxins as they lack of anatomical structures to use the toxins and feed on nematodes. An example is given by *Conocybe lactea*, which produces a paralizing toxin for rabditoids or fungivorous nematodes of the genus *Aphelenchoides*, but the nematode is not used as food source (Hutchison et al., 1996). The fungus *Myrothecium verrucaria* strain X-16 can prey eggs, second stage juveniles and adults of *M. hapla*, probably through the production of proteases, chitinases and toxins (Dong et al., 2015). Other fungi, such as *Drechmeria coniospora*, *Harposporium anguillulae*, *Lecanicillium*, *Paecilomyces*, particularly different strains of *Purpureocillium lilacinus* (=*Paecilomyces lilacinus*) etc., have shown predatory ability. Among them *Paecilomyces* spp. are able to prey fungi (Bordallo et al., 2002; Moreno-Gavíra et al., 2020), nematodes and arthropods (Thies and Grossman, 2006; Kiewnick and Sikora, 2006) and, therefore, contribute to limit damages cause by the synergism of fungi and nematodes (Ragozzino and d’Errico, 2011). The entering into the host or secretion of enzyme complexes occurs after their contact with the prey, mechanically or enzymatically. The mechanical penetration is by appressoria (Khan et al., 2006), while the enzymatic is through the release of cellulase, glucanase, laccase, leucinoxine, lipase, pectinase, protease, and chitinase or xylanase (Prabavathy and Valli, 2012). *P. lilacinus* produce proteolitic enzymes (protease and chitinase) capable of rapidily degrading the cuticle of nematodes in which they enter especially in eggs, juvenile stages and adults of different genera of nematodes by spore germination, further ramification of the hypha and formation of appressoria (Khan et al., 2006).

##### 2.1.4.6 Mycorrhizal fungi

Mycorrhizal fungi live in symbiosis with higher plants favouring the plant uptake of water and nutrients, while the plant, because of the photosynthesis activity, produces organic molecules, as sugars or lipids, available to the fungus. More than 80% of the plants establishes symbiotic relationshis with arbuscular mycorrhizal fungi (AMF) (Brundrett and Tedersoo, 2018); the only exceptions are members belonging to the botanical families Chenopodiaceae, Brassicaceae and Proteaceae (Manzoni et al., 2012). Among the AMF the most representative species are: *Rhizophagus intraradices* (=*Glomus intraradices*) and *Funneliformis mosseae* (=*G. mosseae*). Unfortunately, in this symbiotic association, some factors may limit the AMF colonization of the plant roots. Among them there are: biotic (Faghihinia et al., 2020) and abiotic (Lenoir et al., 2016) stresses; the frequent monoculture and continuous soil tilling (Peng et al., 2024); the extensive use of synthetic fertilizers (Babalola et al., 2022); use of pesticides (Hage-Ahmed et al., 2019; Pagano et al., 2023), especially fungicides (Duley and Boselli, 2022), which are the cause of low diversity and abundance of AMF. Nevertheless, the effects of this association is crucial in improving of up to 50% plant uptake of nitrogen and up to 90% of phosphorous, plus potassium and a variety of micro-elements (You et al., 2021) and water (Garcia et al., 2016). Moreover, because of phytohormone regulation in plants (Begum et al., 2019) and the improved chlorophyl synthesis occurs a better plant development (Baslam et al., 2013). In turn, plants beside affecting the secondary metabolic activity of the vegetal cells, active resistance mechanisms (Vos et al., 2012) and exert an antagonistic or inhibitory effect to the soil-borne parasites (Berg, 2009), leading to reduced damage to plants by a number of diverse parasites (fungi, nematodes, bacteria, etc.).

However, besides these positive effects, it must be aware that plants and their associated AMF utilize the same nutrients, particularly nitrogen, and that, therefore, they may compete each other. Results of such interaction, depending on the context in which they occur, may turn to parasitism (Hoeksema et al., 2010; Johnson et al., 2015). It has also been shown that AMF may affect competitive interactions, on growth and uptake of nutrients in the tissues of host and non-host vegetal species (Bahadur et al., 2023). In practice, because of a number of variables, the results from field applications of AMF indicate that often the estimated parameters are not correlated to the crop performance (Lehman et al., 2019).

*Trichoderma* spp. are mostly asexual Ascomycete fungi that colonize all environments, overall decomposing organic matter. This genus can feed also on others fungi commonly isolated from the soil in which they live as saprophytes, playing an important role in the degradation of vegetal polysaccarids. *Trichoderma* have a well recognized ecological role as they interact not only with pathogenic microorganisms but also with the entire soil microbioma (Harman et al., 2004). The success of its soil colonization and, therefore, as BCAs is due to its ability to increase rapidly in different substrates, to flourish despite competition for nutrients, space, and light (Harman, 2006; Vinale et al., 2008), and to survive in the presence of many toxic chemical substances, such as fungicides, herbicides, and other polluting organic compounds (Kamala et al., 2015; Zin and Badaluddin, 2020; Escudero-Leyva et al., 2022). For instance, it has been demonstrated that T. viride degrades trinitrotoluene (TNT) (Alothman et al., 2020) and *T. inhamatum* reduces the concentrations of hexavalent chromium (Morales-Barrera and Cristiani-Urbina, 2008). *Trichoderma* are the largest component of the microbiome in different soil ecosystems (agricultural fields, grasslands, forests, deserts, etc.) of various climatic zones. They have important ecological roles and are the active ingredient of more than 60% of the control bioformulations registered at the world level (Abbey et al., 2019). *Trichoderma* can parasitize a large number of fungi, involving many lytic enzymes used to degrade cell walls and chitin for alimentary purposes, to recover the cell wall during aging, and its autolysis for remodeling during development and hyphal branching (Gruber and Seidl-Seiboth, 2012). *Trichoderma* secondary metabolites (SMs) (Marra et al., 2019; Marra et al., 2020) may reduce the severity of fungal and bacterial plant diseases (Papaianni et al., 2020; Villegas et al., 2024) and of phytophagous (nematodes and insects) (d’Errico et al., 2021; Anwar et al., 2023) utilizing different mechanisms (competition, antibiosis, and direct mycoparasitism), promotion of plant growth, and induction of resistance in plants (Shores et al., 2010; Villao-Uzho et al., 2024). Important is also the induction of resistance due to chemical products (elicitors) released by the cell walls of the host plant (endoelicitors) and the infecting microorganism (esoelicitors) (Saravanakumar et al., 2016). The involved mechanism is often typical of the fungal strain and depends on the kind of interaction established between the antagonistic microorganism, the pathogen, and the host plant (Benítez et al., 2004; Ghorbanpour et al., 2018). It has been observed that root exudates (RE), obtained from plants grown in a split root system and exposed to different abiotic stresses, stimulate growth and act as chemoattractants for the fungus (Lombardi et al., 2018). Toward nematodes, *Trichoderma* act directly by parasitizing eggs and J2s and indirectly through the production of hydrolytic enzymes and secondary metabolites that destroy the nematode cuticle (De Oliveira et al., 2021). The activity of the enzyme protease in degrading the eggshell, allowing penetration and colonization, is more important than chitinase (Swe et al., 2011). Afterwards, it has been demonstrated that *T. asperellum* and *T. harzianum* can penetrate even the gelatinous matrix of the egg mass enveloping the eggs of the RKN *Meloidogyne javanica*, suppressing egg hatch (Ibrahim et al., 2020). The parasitism of *Trichoderma* is due to several mechanisms used to facilitate crossing the nematode cuticle or the eggshell (Kerry and Hominick, 2002; Hallman et al., 2009). Microscopy observations elucidated the different steps of *Trichoderma* to parasitize eggs. They consist of hyphal wrapping of the eggs, formation of appressoria-like structures, and hyphae that enter and develop inside eggs, later producing conidiophores that move toward contiguous eggs (Coloma et al., 2016). The success of parasitism by *Trichoderma* requires mechanisms to facilitate entry through the nematode cuticle or the eggshell (Hallman et al., 2009). Moreover, as observed in plants inoculated with *T. harzianum* BI, the beneficial effects of the enzymes inducing plant resistance, such as peroxidases (POX), polyphenol oxidases (PPO), and phenylalanine ammonia lyase (PAL), should not be neglected, as they increase significantly. Based on these findings, it can be concluded that direct parasitism of the eggs and induction of resistance are the two main mechanisms *T. harzianum* BI utilizes to suppress the development of *M. javanica* (Sahebani and Hadavi, 2008). Other results show that *T. asperellum* and *T. harzianum* have potential for use in biological control of *Pratylenchus brachyurus* in soybeans (De Oliveira et al., 2021). Also, in *B. xylophilus*, it has been observed that chitinases GH18 genes occurring in this nematode are superior to those occurring in RKNs, affecting the life cycle and specifically leading to reduced egg-laying and a delay in egg hatching (Ju et al., 2016). Recent studies have ascertained that some species, such as *T. atroviride*, produce several metabolites (epipolytiodioxopiperazine (ETP), peptaibols, steroids, lactones, trichothecenes, and others) that act similarly to synthetic pesticides (Khan et al., 2020a; Khan et al., 2020b). Toward synthetic pesticides, *Trichoderma* has a negative impact; however, some, at low concentrations, can be used in combined applications (Korsten and Jeffries, 2000).

#### 2.1.5 Yeasts

They are eukaryotic unicellular organisms estimated to represent 1% of the described fungal species (Kurtzman, 2011). Generally, they prefer neutral subacid soil and rather warm temperatures, but they can also be found everywhere, including very acidic, alkaline, and cryogenic soils. Moreover, some isolates also survive in extreme environmental conditions, such as low moisture content, poor nutrients, UVA, osmotic and oxidizing stress, and toxic compounds. The resistance to adverse conditions is ideal for yeasts used in biocontrol strategies (Sui et al., 2015; Péter et al., 2017; Yurkov, 2017; Buzzini et al., 2018).

The most abundant yeasts in the soil are those belonging to the genera *Cryptococcus* and *Trichosporon*. According to a new systematic classification (Liu et al., 2015), the soil-inhabiting species of the genus *Cryptococcus* have been transferred to the genera *Goffeauzyma, Heterocephalacria, Hannaella, Holtermaniella, Naganishia, Papiliotrema, Piskurozyma, Saitozyma*, and *Solicococcozyma*, while those of the genus *Trichosporon* have been transferred to the genera *Apiotrichum, Cutaneotrichosporon*, and *Tausonia.* The numerous species of *Saccharomyces*, in particular *S. cerevisiae*, known as baker’s yeast or brewer’s yeast, prevails and are commonly found in soils. Generally, *Saccharomyces* are transitory species into the soil, as they reproduce on substrates above the soil, mainly fruits (Sampaio and Gonçalves, 2017). Besides other properties, yeasts have beneficial effects on both plant growth and protection against several plant parasites.

Most of the investigations on the effects of yeasts on the control of PF have been conducted under *in vitro* conditions; they have highlighted that several yeasts are effective in limiting the populations of various PF, especially in post-harvest (Kheireddine et al., 2021; Palmieri et al., 2021; Amjad Ali et al., 2024), a period during which agricultural products may suffer yield losses of 10-30% (Aidoo, 1993). Because of their efficacy in controlling plant parasitic fungi, yeasts are also beneficial in reducing mycotoxins in plant products (Pfliegler et al., 2015; Oztekin et al., 2023). Several yeasts have been selected as BCAs and can be easily used as active ingredients in bioformulations (Droby et al., 2009; Wisniewski et al., 2016; Mukherjee et al., 2020). Yeasts do not produce toxic compounds, and their mode of action is based on their rapid colonization of injured fruits, competition with the pathogenic agent for space and nutrients, secretion of lytic enzymes to degrade the cellular walls of fungi (Spadaro and Droby, 2016; Al-Qaysi et al., 2021), induction of resistance to stresses in the host plants, production of biofilms on vegetative tissues (Scherm et al., 2003), and modification of beneficial plant secondary metabolites (Pastore et al., 2020; Wang et al., 2021).

Yeasts, especially *S. cerevisiae*, have also been shown to be very effective in significantly controlling PPNs, in particular several species of RKNs (*M. incognita, M. javanica*, etc.) (Noweer and Hasabo, 2005; Karajeh, 2013; Osman et al., 2020; Guadalupe-Daqui et al., 2023). The action of *S. cerevisiae* on nematodes depends on the ability of fungi to transform sugars into ethyl alcohol and CO_2_, which are toxic to nematodes, resulting in increased crop disease severity (Siddiqui et al., 2012). Recently, the yeast *P. terrestris* strain PT22AV have also shown its nematicidal potential in the management of *M. incognita* (D’Addabbo et al., 2024).

#### 2.1.5 Viruses

The associations between nematodes and viruses are characterized by severe damages on numerous crops. The species capable of transmitting different plant viruses are nematodes included in the families of Longidoridae (Dorilaimida) and Trichodoridae (Triplonchida). Among Longidoridae family, species of the genera *Xiphinema*, *Longidorus*, and *Paralongidorus* transmit *Nepovirus* and *Sadwavirus*; whereas, among the Trichodoridae, the species of the genera *Trichodorus* and *Paratrichodorus* are vectors of *Tobravirus*. The first plant virus found to be transmitted by nematodes has been the Grapevine fanleaf virus (GFLV). This is the most economically important grape virus, and it is vectored by the nematode *X. index* (Hewitt et al., 1958). This nematode is also among the most well-known and world-wide spread (Brown et al., 1995), and the virus may lead to plant death (Nicholas et al., 2007). *X. index* is rather common and frequently found in large soil population densities (d’Errico et al., 2024). Later on, thanks also to genome sequencing, new viruses and their hosts have been isolated and identified. It has been shown that a number of new viruses are associated with a variety of soil organisms, including nematodes other than those reported as virus vector species (Vieira and Nemchinov, 2019; Vieira et al., 2020, 2022).

Unfortunately, information on viruses parasitic to nematodes is very poor. However, some viruses parasitic to the model nematode *Chaenorhabbditis elegans* have been identified and are being studied (Felix et al., 2011; Frézal et al., 2019). Moreover, very interesting are studies on the use of tobacco mild green mosaic virus (TMGMV) as a nanocarier for nematicides (Chariou and Steinmetz, 2017).

## 3 Discussion

Soil microorganisms, especially bacteria and fungi along with nematodes, play a fundamental role in soil life, increasing soil fertility and thus improving plant growth and health. Several of them are parasitic on nematodes, while many others may strength the plant and also activate the plant natural resistance to them, thus resulting in better growth and yield of crops. This biotic component occurs in all kinds of ecosystems but is richest in the plant rhizosphere. The associations can involve multiple partners, may result in competition, predation, parasitism, and mutualism, and play an important role in the ecosystem, especially in maintaining its equilibrium (Topalovic and Heuer, 2019). Rich plant diversity and the biotic components of the soil improve water penetration and storage, reduce soil erosion, enhance plant nutrition, decrease populations of phytopathogenic organisms, and stimulate soil organic matter recycling. If the soil is left undisturbed all soil micro-organisms reach an equilibrium in which the population density of each of them, if parasitic to plant, do not cause severe damages. Therefore, maintaining good biodiversity is instrumental for the eco-sustainability of agricultural production (Mocali et al., 2015a; Mocali et al., 2015b; Lazzaro et al., 2018; Landi et al., 2020; Landi et al., 2022; Landi et al., 2023; Maccherini et al., 2021; Giffard et al., 2022; Del Duca et al., 2024). This is why the biodiversity is considered the heartbeat of life on Earth (Chiarucci et al., 2024). Unfortunately, biodiversity richness is jeopardized by agricultural practices, especially soil tilling, lack of sound crop rotations, and the extensive use of fertilizers and synthetic pesticides (Wachira et al., 2014). Soil tilling is a useful practice in agriculture to prepare the soil for sowing, control weeds, and manage various pests and pathogens. Depending on their intensity, soil tilling has shown significant disturbance of the soil microbiota, which can be restored if the soils are left fallow, preventing soil erosion, washing away of nutrients, and the release of greenhouse gases into the atmosphere (Frøslev et al., 2022). Crop rotation is a basic technique for sustainable agriculture, thanks to multiple beneficial effects overall based on: enhanced soil fertility because the removal of nutrients is diversified according to the needs of the different rotated crops, thus managing nutrients in the soil effectively; managing phytophagous and pathogenic organisms by interrupting their life cycles and reducing the need for synthetic pesticides; and reducing soil erosion (Shah et al., 2021; Sekhar et al., 2024). The excessive use of nitrogen and other macroelements (phosphorus and potassium) to increase agricultural yields has reduced biodiversity (Shen et al., 2010; Lin et al., 2012; Liang et al., 2015), modified the composition and structure of microbial communities (Stevens et al., 2004; Huang et al., 2019), particularly fungi (Wang et al., 2011), changed soil structure (Vitousek et al., 1997; Rousk et al., 2011), and affected plant growth (Clark et al., 2007). The abuse of synthetic pesticides used to control plant parasites and infesting weeds are of worldwide concern because of their negative impact on soil biodiversity (Bernhardt et al., 2017; Wang et al., 2020; Pelosi et al., 2021) and for environmental pollution (Arya et al., 2021; Beaumelle et al., 2023). Biocides have negative effects on the biodiversity of plant species, carabidae, and birds that make their nests in agricultural fields, as well as on the potential biological control of parasites (Geiger et al., 2010). Of particular concern are fumigants, the use of which causes a devastating biological vacuum in the soil treated with them; however, this may become beneficial if these soils are later treated with biological agents that are favored in their soil colonization (d’Errico et al., 2023). Climatic changes may also cause deep modifications in ecosystems, especially in phytophagous organisms, phytopathogens, and infesting weeds (Fand et al., 2012; Muluneh, 2021; Wang et al., 2021; Moerman et al., 2023; Nnadi et al., 2021; Williams et al., 2024). The main modifications refer to organisms, their species, and reproduction and survival rates, which have led to the extinction of species in some areas (Parmesan, 2006). It would be desirable to implement management tactics that reduce soil pathogenic organisms while increasing beneficial ones. Unfortunately, this remains a very difficult task although some imputs could, at least partially, fulfil this gap. This author feels that using little impacting crop management tactics, such as crop rotations, non chemical fertilizers (manure and green manure), addition of beneficial organisms (PGPR, mychorrizal fungi) would improve soil biodiversity favourable to plant growth and yield increase. According to Jacobs et al. (2019), in Europe,be following changes in temperature, rainfalls, and other extreme meteorological and climatic events, continuous and more negative impacts are expected on crop yields and livestock. Also, Gullino et al. (2021) analyzed the geographical distribution of different plant parasites and presented examples of the effects of climate change on plant pests in different climate zones. These authors show that climate changes will result in increasing plant health problems, and modifications in plant protection strategies.
